# Recess physical activity and school-related social factors in Finnish primary and lower secondary schools: cross-sectional associations

**DOI:** 10.1186/1471-2458-14-1114

**Published:** 2014-10-28

**Authors:** Henna L Haapala, Mirja H Hirvensalo, Kaarlo Laine, Lauri Laakso, Harto Hakonen, Anna Kankaanpää, Taru Lintunen, Tuija H Tammelin

**Affiliations:** LIKES – Research Center for Sport and Health Sciences, Jyväskylä, Finland; Department of Sport Sciences, University of Jyväskylä, Jyväskylä, Finland

**Keywords:** Physical activity, Recess, School, Social factors, Peer relationships, Relatedness, School climate, Children, Adolescents

## Abstract

**Background:**

Participation in physical activities provides students with opportunities for social interaction and social skills development. The purpose of this study was to investigate the associations of students’ recess physical activity with school-related social factors.

**Methods:**

Data were collected in 19 schools countrywide in autumn 2010, and 1463 students from grades 4 and 5 (primary school) and from grades 7 and 8 (lower secondary school) completed an anonymous questionnaire. Multiple linear regression analysis was used to investigate whether self-reported physical activity at recess was associated with peer relationships at school, relatedness to school and school climate. Analyses were adjusted for self-reported overall physical activity and conducted for primary and lower secondary schools. Multi-group analysis was used to test sex differences among the associations.

**Results:**

In primary school, physical activity at recess was positively associated with peer relationships at school (boys: b = 0.17, p = 0.007 and girls: b = 0.21, p <0.001), relatedness to school (boys: b = 0.18, p = 0.002 and girls: b = 0.24, p <0.001) and school climate (girls: b = 0.17, p = 0.001), after adjusting for overall physical activity. In lower secondary school, physical activity at recess was positively associated with peer relationships at school (boys: b = 0.09, p = 0.006 and girls: b = 0.12, p = 0.010) but not with other school-related social factors. No sex differences were observed in these associations.

**Conclusions:**

Our results suggest that students’ participation in physical activities during school recess is positively associated with students’ school-related social factors. In the future, it would be worthwhile to study how physical activity at recess should be organised in order to support the development of school-related social factors.

## Background

Physical activity has multiple benefits for the health and well-being of school-aged children and adolescents [1]. Studies have also linked physical activity to learning, cognitive functions and academic performance [2, 3]. Aside from these benefits, there is some evidence that various physical activity settings can improve social development through opportunities for social interactions, meeting friends, co-operating with others, and problem solving in children and adolescents [4–6]. The recent emphasis on the promotion of school-aged children’s physical activity in the school setting [7, 8] also raises the question if school-time physical activity might benefit students’ school experiences and their school-related social factors.

Recess is an important arena for learning social skills and experiencing social life; recess provides children with the opportunity to engage in free play, and this may improve their social development [9]. During recess, students have the opportunity for informal social interaction without the structure of the classroom and adult control, and through play they learn the skills needed in everyday life, including negotiation, problem solving, and co-operation [10]. It has also been suggested that these interactions and the skills acquired during recess may contribute to students’ school experience [11].

The multiple definitions of students’ relationship and connection to school tend to concern the same themes: belonging, liking school, support from teachers and peers, engagement in academics, fair discipline, and participation in extracurricular activities [12]. In this study, the outcome measures of school-related social factors included peer relationships at school, relatedness to school, and school climate. *Peer relationships at school* consist of e.g. getting help from classmates, getting along with schoolmates, and having friends in school [13, 14]. *Relatedness to school* refers to the feeling of being supported and safe amongst others [15, 16]. *School climate* is usually a combination of multiple dimensions, such as teacher and peer support, student autonomy, and clarity and consistency in school rules [17]. In addition, the quality of social life at schools is connected to sex and age; girls reported higher school-related social support than boys, and these perceptions deteriorated with increasing age in both sexes [17–20].

In general, physical activity situations seem to provide fruitful settings for social interactions and create encounters between participants [6, 21]. Because sport and physical activities bring people together in a shared interest, they may build co-operative experiences and strengthen their sense of cohesion. These experiences then may result in improved social capital and promote social inclusion [22, 23]. For example, Iannotti and associates observed that higher overall physical activity was related to better peer relationships in adolescents at the ages of 11, 13 and 15 [24]. Within the school day, one of the key opportunities for students’ physical activity is recess [25]. Playground games have a largely social function for younger students, and the games are a key element in break-time activities until age 11 [5]. Physical education and sports in schools have also been linked to students’ improved social development, social skills, and prosocial behaviour [4, 21]. Schools that provide opportunities for physical activities at recess could offer practical means to socialise with new peers outside the structure of the classroom to adolescents in the school transition phase and in so doing, enhance their learning, motivation, academic outcomes [26–29] and liking of school [30]. However, few studies have examined the associations of recess physical activity with social factors in the school setting.

The aim of this study was to investigate the cross-sectional associations of recess physical activity with peer relationships at school, relatedness to school and school climate among boys and girls in grades 4–5 (primary school) and 7–8 (lower secondary school). In addition, we evaluated whether these associations differed between boys and girls. We hypothesise that participation in physical activities at recess is positively associated with these school-related social factors.

## Methods

### Study design and population

This study used cross-sectional data collected as a part of the evaluation of the national Finnish Schools on the Move action programme, more specifically from the programme’s pilot phase 2010–2012. The aim of the programme is to establish a physically active culture in Finnish comprehensive schools, but the programme schools had not yet started promotion activities at the time of the data collection. The programme and its design is described in an earlier article by Haapala et al. [31].

Cross-sectional data were collected from 26 programme schools in grades 1 to 9 in autumn 2010. Youngest students in grades 1 to 3 were excluded from this study due to the different questionnaire used with them. Altogether 19 schools with students from grades 4 to 5 and grades 7 to 8 were included in this study. Seven schools were located in an urban area, one school in a suburban area and 11 schools in a rural area. The response rates were 91% in grades 4 and 5 and 83% in grades 7 and 8. The study population consisted of 579 children in grades 4 and 5 (primary school; mean age 11.0 ± 0.6; 53% boys) and 884 adolescents in grades 7 and 8 (lower secondary school; mean age 14.1 ± 0.6; 49% boys). The languages most often spoken at home were Finnish (83%), Swedish (12%) and other languages (5%). During their regular school day, students completed an anonymous self-report questionnaire with measures of study variables and demographic items (e.g. sex, age and grade). Researchers administered the questionnaire in the classroom, checked that students understood it and answered possible questions.

### Ethics statement

The study protocol and consent procedure were approved by the Ethics Committee of the University of Jyväskylä, and all measurements were carried out in accordance with the Declaration of Helsinki [32]. At the start of the study, the participants were informed of the study protocol and the purpose of the study in two ways: verbally and in writing on the participant information sheet on the cover page of the questionnaire. Participation was voluntary and no individual identification information was collected. The participants gave their assent/consent to participate in the study by returning the questionnaire to the researchers. The consent of the participants’ parents was not obtained. According to the Ethical principles of the Finnish National Advisory Board on Research Ethics, it is not necessary to request a guardian’s permission in Finland if the head teacher of a school has determined that the study would produce useful information for the school and the study can be carried out as part of the normal activities of the school. Broad questionnaires that do not directly collect identifying information for research purposes can be carried out without the consent of parents [33].

### Recess in the Finnish school system

In the Finnish school system, the lengths of recesses and lunch breaks are not regulated by national law. Thus, in practice students in Finland are provided with several recess periods daily, and schools arrange lessons and break times relatively independently. In primary school (grades 1–6) and lower secondary school (grades 7–9), there are usually two to four recess periods of 10–15 minutes after each 45–90 minute lesson and one longer recess period of 30 minutes for school lunch and other activities.

### Physical activity

*Physical activity at school recess* was measured with a scale of physical activities at recess. The question, “What do you usually do at school recess?” was followed by five statements: “I sit”, “I stand around”, “I walk”, “I take part in physically active play”, and “I play ball games, for example football”. Students responded on a 4-point scale (0 = never; 1 = sometimes; 2 = at most recesses; and 3 = at all recesses). To create a new variable, “physical activity at recess”, the items of sitting and standing were reverse coded, and the items in this measure were averaged. The internal reliability (Cronbach’s α) for this new measure was acceptable (α = 0.62).

In order to evaluate the validity of measures of recess activities, a separate smaller subsample in a different dataset from the Finnish Schools on the Move programme was used to compare these self-reported measures with objectively measured school-day physical activity. Two-hundred and twenty-nine students from grades 4-5 and 7-8 (42% boys) completed identical questionnaires to this study and wore ActiGraph GT1M or GT3X -accelerometers for seven consecutive days in autumn 2010. Recess activity measures were then compared with objectively measured school day sedentary time, light physical activity and moderate- to vigorous-intensity physical activity (MVPA, minutes/hour). Objectively measured school day MVPA had strong positive associations with physically active play, ball games, and physical activity at recess, and there were strong inverse associations with sitting and standing around (Table 1). Walking correlated inversely with the objectively measured school day sedentary time in all the students and in boys.

**Table 1:** **Pearson’s correlation coefficients for self-reported physical activities at recess and objectively measured physical activity (minutes/hour) during the school day in grades 4-5 and 7-8 students**

	All (n = 229)			Boys (n = 95)			Girls (n = 134)		
	Sed	Light PA	MVPA	Sed	Light PA	MVPA	Sed	Light PA	MVPA
Sitting	0.31**	-0.31**	-0.40**	0.41**	-0.32**	-0.44**	0.31**	-0.42**	-0.42**
Standing around	0.45**	-0.46**	-0.50**	0.47**	-0.34**	-0.53**	0.44**	-0.53**	-0.47**
Walking	-0.18*	0.14	0.06	-0.22*	0.20	0.20	-0.15	0.08	-0.09
Physically active play	-0.57**	0.57**	0.52**	-0.63**	0.53**	0.61**	-0.55**	0.63**	0.42**
Ball games	-0.51**	0.48**	0.57**	-0.62**	0.50**	0.62**	-0.43**	0.44**	0.47**
Combined measure									
PA at recess^1^	-0.57**	0.55**	0.59**	-0.63**	0.51**	0.64**	-0.56**	0.61**	0.51**

Note: Sed = sedentary time. PA = physical activity. MVPA = moderate- to vigorous-intensity physical activity. PA = physical activity. *Correlation is significant at the 0.05 level. **Correlation is significant at the 0.01 level. ^1^ Measure is the mean of the measures “sitting”, “standing around”, “walking”, “physically active play”, and “ball games”.

*Overall physical activity* was used to adjust for the possible effects of overall activity on being physically active at recess. The level of overall physical activity was evaluated with a question taken from WHO’s Health Behaviour in School-aged Children (HBSC) survey [34]. The measure has been reported to be reliable (intraclass correlation coefficients ranging between 0.77 and 0.82) [35, 36] and valid (significant correlation with accelerometer data: r = 0.40, p <0.001) [36] among school-aged children. Students used an 8-point scale (0–7 days) to report the number of physically active days they had had during the previous week with at least 60 minutes of MVPA per day.

### School-related social factors

*Peer relationships at school* were measured with a subscale of the social relationships domain from the School Well-being Profile developed according to the School Well-being Model [13, 14, 37]. The scale included eight statements: “Pupils in my class enjoy being together”, “Working in teams goes well in my class”, “Classmates interrupt if some pupil is being bullied”, “Classmates help each other in school tasks”, “Classmates help each other in problem situations”, “It’s easy to get along with schoolmates”, “I have friends at this school”, and “Schoolmates accept me as I am.” Students responded on a 5-point Likert scale (0 = totally agree; 1 = agree; 2 = neither agree nor disagree; 3 = disagree; and 4 = totally disagree). The items in this measure were averaged, and the internal reliability (Cronbach’s α) for the scale in this study was high (α = 0.85). Additionally, the structure of the questionnaire has been verified to fit the School Well-Being Model and the internal reliability (Cronbach’s α) of the social relationships subscale was good (α = 0.79 in primary schools, α = 0.89 in lower secondary schools) [13].

*Relatedness to school* was measured with a 5-item subscale from the Need for Relatedness Scale [15]. This scale was originally developed for the workplace, but its construct validity and reliability have also been supported in physical education settings [38, 39]. The stem was modified for the school context to read, “In this school, I feel…”, followed by items of feeling “supported”, “listened to”, understood”, “valued” and “safe”. Students responded on a 5-point Likert scale (0 = totally agree; 1 = agree; 2 = neither agree nor disagree; 3 = disagree; and 4 = totally disagree). The items in this measure were averaged, and the internal reliability (Cronbach’s α) for this scale was high (α = 0.92).

*School climate* was measured with a question modified from the home climate question in HBSC surveys [40] by replacing the word “home” with “school”. Students answered the following question: “How do you experience the climate in your school?” Students responded to the question on a 5-point scale (0 = very good; 1 = fairly good; 2 = not good or poor; 3 = fairly poor; and 4 = very poor).

All the measures of school-related social factors were reverse coded before the analyses, so that higher scores indicated positive measures.

### Statistical analyses

Statistical analyses were conducted using Mplus statistical package version 7.0 [41]. The level of significance was set at 0.05. Means and standard deviations were calculated for continuous variables for four student groups (boys and girls in grades 4–5 and in grades 7–8). A Student’s *t* -test was used to investigate the differences in physical activities and school-related social factors between the aforementioned student groups. Pearson’s correlation coefficients were calculated for the study variables.

Multiple linear regression analyses were conducted to discern whether *physical activity at recess* and *overall physical activity* were associated with *peer relationships at school*, *relatedness to school* and *school climate* in primary school (grades 4–5) and in lower secondary school (grades 7–8). To handle non-independence of the data due to clustered sampling, with school as the primary sampling unit, the special analytical feature of Mplus for complex sample data (command TYPE = Complex) was used. This approach produces corrected standard errors for regression coefficients by using a sandwich estimator in the computations.

The intra-class correlation coefficients (ICC) were calculated for the outcome variables in primary and lower secondary schools in order to evaluate the proportion of the total variance explained by the cluster-level. The distributions of all school-related social measures were negatively skewed and leptokurtic. In addition, the distribution of physical activity at recess was positively skewed and overall physical activity was negatively skewed. Maximum likelihood estimation with robust standard errors (MLR) was used when the assumption of normality was not met.

Multiple linear regression models were fitted for boys and girls by using multiple-group analysis method. Analyses were conducted separately for primary and lower secondary schools. First, models predicting the school-related social factors by physical activity at recess and overall physical activity were fitted without equality constraints across sex groups (saturated model). After that, the regression coefficients of physical activity at recess and overall physical activity were estimated as equal across sex groups (more restricted model) in order to test whether there were sex differences in the associations. The Satorra-Bentler scaled χ^2 -^ difference test was used to evaluate whether the more restricted model was rejected in favour of the saturated model. When the difference test produced significant loss-of-fit, the effects of physical activity (overall physical activity or physical activity at recess) on school-related social factors were interpreted to differ between boys and girls. When sex differences were observed, the equality of each regression coefficient was tested for significance by using the model constraint feature in Mplus. Standardized estimates, standard errors and *p*-values were reported, as well as the coefficient of determination (R^2^) for the saturated models. Full information maximum likelihood (FIML) estimation was used under the assumption of data missing at random (MAR) in analysing incomplete data.

## Results

### Descriptive statistics

We observed two trends in how students reported their participation in physical activities at recess and their overall physical activity (Table 2). The first trend was that the levels of physical activity at recess were higher among boys than among girls in primary and lower secondary schools. The second trend was that for both boys and girls physical activity at recess was more common in grades 4 to 5 than in grades 7 to 8. In overall physical activity, the trends were similar; boys and younger students also had more often at least 60 minutes of MVPA compared to girls and older students. All school-related social factors – peer relationships at school, relatedness to school, and school climate – were reported more positive in grades 4 to 5 than in grades 7 to 8, but were not shown to be different between boys and girls (Table 2).

**Table 2:** **Descriptive statistics of the study variables**

	Primary school			Lower secondary school			Between	
	(Grades 4-5)			(Grades 7-8)			School levels	
	Boys (n = 308)	Girls (n = 271)	p for sex	Boys (n = 436)	Girls (n = 448)	p for sex	p for boys	p for girls
Different physical activities at recess (range 0-3)								
Sitting (n)	250	230		402	431			
Mean, SD	0.62, 0.69	0.73, 0.60	0.072	1.82, 0.70	1.70, 0.69	**0.009**	**<0.001**	**<0.001**
Standing around (n)	254	235		383	434			
Mean, SD	1.22, 0.93	1.28, 0.79	0.440	1.70, 0.76	1.79, 0.68	0.085	**<0.001**	**<0.001**
Walking (n)	264	246		336	417			
Mean, SD	1.91, 0.93	2.17, 0.78	**0.001**	1.59, 0.85	1.59, 0.78	0.938	**<0.001**	**<0.001**
Physically active play (n)	269	246		356	398			
Mean, SD	1.81, 0.98	1.49, 0.85	**<0.001**	0.75, 0.80	0.39, 0.64	**<0.001**	**<0.001**	**<0.001**
Ball games (n)	259	232		352	394			
Mean, SD	1.85, 0.99	1.06, 0.95	**<0.001**	0.56, 0.76	0.21, 0.45	**<0.001**	**<0.001**	**<0.001**
PA at recess^1^ (range 0-3) (n)	303	267		433	448			
Mean, SD	1.99, 0.50	1.79, 0.50	**<0.001**	1.08, 0.54	0.96, 0.40	**<0.001**	**<0.001**	**<0.001**
Overall PA (range 0-7)								
Days per week with ≥60 min MVPA (n)	297	256		425	429			
Mean, SD	4.98, 2.02	4.55, 1.87	**0.010**	4.53, 2.00	4.13, 1.77	**0.002**	**0.003**	**0.003**
School-related social factors (range 0-4)								
Peer relationships at school (n)	285	257		403	431			
Mean, SD	3.01, 0.68	3.02, 0.65	0.909	2.81, 0.62	2.82, 0.57	0.747	**<0.001**	**<0.001**
Relatedness to school (n)	293	257		415	440			
Mean, SD	3.05, 0.83	3.04, 0.85	0.883	2.76, 0.76	2.82, 0.73	0.239	**<0.001**	**0.001**
School climate (n)	304	266		425	445			
Mean, SD	3.12, 0.72	3.11, 0.75	0.970	2.80, 0.78	2.84, 0.65	0.356	**<0.001**	**<0.001**

Note: ^1^Measure is the mean of the combined measures “sitting”, “standing around”, “walking”, “physically active play”, and “ball games”. PA = physical activity. MVPA = moderate- to vigorous-intensity physical activity. SD = standard deviation. P-values from Student’s *t*-test. Statistically significant values (p < 0.05) are presented in bold.

According to the Pearson’s correlation coefficients, peer relationships at school, relatedness to school, and school climate were associated with one another in both school levels in both sexes (Table 3). In primary school, physical activity at recess was associated with all three school-related social factors and overall physical activity in both sexes. In lower secondary school, physical activity at recess was only associated with peer relationships at school in both sexes and overall physical activity in boys. The intraclass correlation coefficients (ICC) for peer relationships at school, relatedness to school, and school climate were in primary schools 0.071, 0.098, and 0.083, respectively, and in lower secondary schools 0.013, 0.023, and 0.072, respectively.

**Table 3:** **Pearson’s correlation coefficients for the study variables for boys (upper triangle) and for girls (lower triangle) in primary and lower secondary schools**

	1	2	3	4	5
Primary school					
1 PA at recess		0.07	0.15**	0.16**	0.12*
2 Overall PA	0.28**		0.15**	0.05	-0.01
3 Peer relationships at school	0.14*	0.03		0.73**	0.35**
4 Relatedness to school	0.20**	0.02	0.71**		0.34**
5 School climate	0.14*	0.00	0.58**	0.58**	
Lower secondary school					
1 PA at recess		0.24**	0.13**	0.09	0.02
2 Overall PA	0.04		0.27**	0.32**	0.12*
3 Peer relationships at school	0.11*	0.13**		0.61**	0.49**
4 Relatedness to school	0.06	0.19**	0.58**		0.51**
5 School climate	0.03	0.11*	0.49**	0.51**	

Note: PA = physical activity. *Correlation is significant at the 0.05 level. **Correlation is significant at the 0.01 level.

### Physical activity at recess and school-related social factors

#### Peer relationships at school

In primary school, physical activity at recess was directly associated with peer relationships at school (boys: b = 0.17, p = 0.007 and girls: b = 0.21, p <0.001) (Table 4). In lower secondary school, physical activity at recess was positively associated with peer relationships at school (boys: b = 0.09, p = 0.006 and girls: b = 0.12, p = 0.010). Sex differences were observed for the models in both primary school (χ^2^ (2) = 6.05, p = 0.049) and lower secondary school (χ^2^ (2) = 26.03, p <0.001). Further analysis revealed that the association between physical activity at recess and peer relationships at school was similar among girls and boys in primary school (b = 0.17 vs. b = 0.21, p = 0.747) and in lower secondary school (b = 0.09 vs. b = 0.12, p = 0.215), but the association between overall physical activity and peer relationships at school differed between sex groups in primary school (b = 0.14 vs. b = -0.03, p = 0.009) and lower secondary school (b = 0.26 vs. b = 0.12, p = 0.006).

**Table 4:** **Multiple liner regression analysis for physical activity (PA) at recess and its association with peer relationships at school, relatedness to school, and school climate**

		Boys			Girls	
	Estimate	SE	p-value	Estimate	SE	p-value
Primary school						
Peer relationships at school						
PA at recess	**0.17**	**0.06**	**0.007**	**0.21**	**0.05**	**<0.001**
Overall PA	**0.14**	**0.06**	**0.020**	-0.03	0.08	0.720
R^2^	0.05			0.04		
Relatedness to school						
PA at recess	**0.18**	**0.06**	**0.002**	**0.24**	**0.05**	**<0.001**
Overall PA	0.04	0.10	0.651	-0.04	0.06	0.525
R^2^	0.04			0.06		
School climate						
PA at recess	0.14	0.08	0.095	**0.17**	**0.05**	**0.001**
Overall PA	-0.02	0.04	0.722	-0.05	0.07	0.546
R^2^	0.02			0.03		
Lower secondary school						
Peer relationships at school						
PA at recess	**0.09**	**0.03**	**0.006**	**0.12**	**0.05**	**0.010**
Overall PA	**0.26**	**0.06**	**<0.001**	**0.12**	**0.03**	**0.001**
R^2^	0.09			0.03		
Relatedness to school						
PA at recess	0.01	0.01	0.667	0.03	0.08	0.739
Overall PA	**0.34**	**0.03**	**<0.001**	**0.18**	**0.08**	**0.017**
R^2^	0.12			0.04		
School climate						
PA at recess	-0.02	0.03	0.493	0.04	0.06	0.434
Overall PA	**0.12**	**0.06**	**0.038**	**0.11**	**0.02**	**<0.001**
R^2^	0.01			0.01		

Note: PA = physical activity. SE = standard error. Standardised regression coefficients are presented. Statistically significant values (p <0.05) are presented in bold.

#### Relatedness to school

In primary school, physical activity at recess was positively associated with relatedness to school among girls and boys (boys: b = 0.18, p = 0.002 and girls: b = 0.24, p <0.001). In lower secondary school, physical activity at recess was not associated with relatedness to school (boys: b = 0.01, p = 0.667 and girls: b = 0.03, p = 0.739). No sex differences were observed for the models (primary school: χ^2^ (2) = 1.73, p = 0.422 and lower secondary school: χ^2^ (2) = 2.34, p = 0.310).

#### School climate

In primary school, physical activity at recess had a positive association with school climate among girls, but the association was not significant among boys (girls: b = 0.17, p = 0.001; boys: b = 0.14, p = 0.095). In lower secondary school, physical activity at recess was not associated with school climate (boys: b = -0.02, p = 0.493 and girls: b = 0.04, p = 0.434). No sex differences were detected for the models (primary school: χ^2^ (2) = 0.24, p = 0.888 and lower secondary school: χ^2^ (2) = 1.40, p = 0.497).

## Discussion

This study addressed students’ physical activity at recess and its connection to social factors emerging in the school setting. Physical activity at recess was positively associated with peer relationships at school in both primary and lower secondary school students, relatedness to school in primary school students, and school climate among girls in primary school. These associations were independent of students’ overall physical activity.

The positive associations of physical activity at recess and peer relationships at school in both school levels supported our original hypothesis. Previous research has identified physical activity as a multidimensional and contextual behaviour [42, 43]; for example, recess activities have been noted to provide opportunities for social development [4, 5]. The provision of recess physical activities could be one factor behind the successful promotion of peer relationships at school and liking school. On the other hand, it should be noted that physical activity at recess could be just one of the many ways to support the positive development of social factors in school. As studies on extracurricular activities have shown, participation in other activities at recess, such as music and other performing arts not included in this study, could have similar benefits for social factors [44, 45]. Furthermore, building social relationships and promoting social responsibility are important learning objectives in school physical education. The Finnish physical education also aims towards positive psychological and social outcomes, such as community spirit, responsibility, fair play, and safety, along with physically active lifestyle [46].

Sport and physical activity in general have been connected to promoting prosocial behaviour and counteracting disaffection in youth, although the mechanisms mediating the effects of sport and physical activity on these positive social outcomes remain unclear [47]. The positive social impact of participation in physical activities, also at recess times, could be increased by providing students with further opportunities to co-operate with each other [47, 48]. However, situations related to physical activity can sometimes have negative effects as well. They may create conflicts between participants, lead to aggressive behaviour and negative peer interaction, and include pressure to make morally questionable choices [6, 45, 49]. In making use of our findings, we emphasise the importance of environmental, contextual, structural, and educational aspects of physical activity situations, such as involving participants in decision making and encouraging them to take leadership roles, in creating positive social experiences [31, 50–52] .

Younger students in this study reported better school-related social factors compared to older students, as reported in other studies [17–20]. The maturation process and onset of puberty causes mental and physical changes [52], and those changes could partly explain the differences observed between younger and older students. In addition, the impact of the transition from primary school to lower secondary school could explain some of the differentiation process in social factors between the age groups. In Finland, transitioning to lower secondary school at age 13 is a major change in students’ lives. For most students, the transition to lower secondary school means new school buildings and teachers as well as many new peers. Different school cultures, new teaching groups, and social norms could affect the perceptions of school-related social factors in older students. A sense of belonging in the school community is one of the basic needs for every student, and it consists of relationships with teachers and peers [16]. These relationships have been found to be resources for positive adjustment during school transition [20]. Our study showed the positive associations between recess physical activity and different school-related social factors; therefore, recess could play a role in providing students with a context to socialise through physical activities, especially in the transition phase.

The results of our study linked students’ physical activity at recess to higher levels of peer relationships at school in both primary and lower secondary school, relatedness to school in primary school and school climate among girls in primary school. Recess would be worth further investigation in the efforts to promote students’ physical activity and social factors in school; recess time offers opportunities for daily physical activity [53, 54], social interactions [55, 56] and adjustment to school [57]. Promoting a physically active school culture could lead to a positive spiral where enhancing a physically active lifestyle in schools benefits the social well-being of students, and this again may support their engagement in physical activity. This approach is also supported by the Finnish national core curriculum for basic education, which emphasises a learning environment supportive of interaction among students, guided towards team-work and a positive atmosphere [46].

The strengths of this study include its relatively large sample of Finnish schoolchildren and high response rates which indicate representative samples within the schools. The analyses also enabled the examination of differences in the associations between the sexes and the adjustment for overall physical activity. Nevertheless, some limitations should be considered when interpreting the findings. Self-report measures, especially of physical activity, can result in measurement errors and social desirability bias [58, 59]. However, the correlations between the self-reported recess physical activity and objectively measured school day physical activity data were moderate. In the future, various types of physical activities at recess should be investigated. Adding more choices to the measure of physical activity at recess, such as dancing, hopping, and skipping, might have detected more recess behaviours in which girls, in particular, participate. In addition, we cannot exclude the possibility that there might be other factors in the students’ backgrounds that may influence physical activity and social factors.

This study is cross-sectional, and it can only indicate associations between measures; it does not suggest causal relationships. Regardless of the possibility of the bidirectional associations, the study results prepare the way for an intervention study to evaluate the effects of increased recess physical activity on school-related social factors. Future research should focus on how physical activity at school recess should be structured to improve students’ social interaction and school experiences. In addition, the evaluation of the long-term effects of these types of interventions are needed.

## Conclusions

The present study showed that physical activity at recess was positively associated with peer relationships at school in primary and lower secondary school students, relatedness to school in primary school students, and school climate among girls in primary school. We encourage further research into how school-based physical activity should be organised to support the development of positive social relationships at school and satisfaction with school.
